# Comparative proteomics reveals the physiological differences between winter tender shoots and spring tender shoots of a novel tea (*Camellia sinensis* L.) cultivar evergrowing in winter

**DOI:** 10.1186/s12870-017-1144-x

**Published:** 2017-11-20

**Authors:** Shengjie Liu, Jiadong Gao, Zhongjian Chen, Xiaoyan Qiao, Hualin Huang, Baiyuan Cui, Qingfeng Zhu, Zhangyan Dai, Hualing Wu, Yayan Pan, Chengwei Yang, Jun Liu

**Affiliations:** 1 Agro-biological Gene Research Center, Guangdong Academy of Agricultural Sciences, Guangzhou, Guangdong 510640 China; 2 Tea Research Institute, Guangdong Academy of Agricultural Sciences, Guangzhou, Guangdong 510640 China; 3 Guangdong Key Lab of Biotechnology for Plant Development, College of Life Science, South China Normal University, Guangzhou, Guangdong 510631 China

**Keywords:** Tea (*Camellia sinensis* (L.) O. Kuntze), Tender shoots, Evergrowing in winter, Proteomics

## Abstract

**Background:**

A recently discovered tea [*Camellia sinensis* (L.) O. Kuntze] cultivar can generate tender shoots in winter. We performed comparative proteomics to analyze the differentially accumulated proteins between winter and spring tender shoots of this clonal cultivar to reveal the physiological basis of its evergrowing character during winter.

**Results:**

We extracted proteins from the winter and spring tender shoots (newly formed two leaves and a bud) of the evergrowing tea cultivar “Dongcha11” respectively. Thirty-three differentially accumulated high-confidence proteins were identified by matrix-assisted laser desorption ionization time of flight mass spectrometry (MALDI-TOF / TOF MS). Among these, 24 proteins had increased abundance while nine showed were decreased abundance in winter tender shoots as compared with the spring tender shoots. We categorized the differentially accumulated proteins into eight critical biological processes based on protein function annotation including photosynthesis, cell structure, protein synthesis & destination, transporters, metabolism of sugars and polysaccharides, secondary metabolism, disease/defense and proteins with unknown functions. Proteins with increased abundance in winter tender shoots were mainly related to the processes of photosynthesis, cytoskeleton and protein synthesis, whereas those with decreased abundance were correlated to metabolism and the secondary metabolism of polyphenolic flavonoids. Biochemical analysis showed that the total contents of soluble sugar and amino acid were higher in winter tender shoots while tea polyphenols were lower as compared with spring tender shoots.

**Conclusions:**

Our study suggested that the simultaneous increase in the abundance of photosynthesis-related proteins rubisco, plastocyanin, and ATP synthase delta chain, metabolism-related proteins eIF4 and protease subunits, and the cytoskeleton-structure associated proteins phosphatidylinositol transfer protein and profilin may be because of the adaptation of the evergrowing tea cultivar “Dongcha11” to low temperature and light conditions. Histone H4, Histone H2A.1, putative In2.1 protein and protein lin-28 homologs may also regulate the development of winter shoots and their response to adverse conditions.

**Electronic supplementary material:**

The online version of this article (10.1186/s12870-017-1144-x) contains supplementary material, which is available to authorized users.

## Background

Tea [*Camellia sinensis* (L.) O. Kuntze] is an important non-alcoholic commercial beverage crop, which has unique aromatic and medicinal properties. Tea tree is a perennial plant, and winter dormancy is its biological adaptation to environmental changes [1, 2]. As temperatures decrease in winter, the tea tender shoots gradually stop growing to form inert buds and enter a period of dormancy. An evergrowing winter tea individual was newly found from a landrace population in Yingde city in the field station of Tea Research Institute, Guangdong Academy of Agricultural Sciences. Unlike other regular tea trees that become dormant, this special individual generates tender shoots (apical bud and associated leaves), enabling it to grow in the winter (January). We propagated this unique individual to a clonal cultivar by grafting the single node cutting, and the evergrowing trait remained stable throughout years. This newly identified evergrowing tea tree cultivar “Dongcha11” can serve as a special resource for studying the physiological mechanism of evergrowing phenotype in winter.

Low temperature and insufficient sunshine during the winter curb the growth of tea buds, which enter dormancy in response to the ecological conditions unsuitable for growth [1, 3]. Many studies have attempted to unravel the mechanism of winter dormancy in tea trees to study the regulation of gene expression [4–7]. Suppression subtractive hybridization analysis of cDNA libraries between dormant and sprouting buds of tea plant showed that the percentage of genes related to the formation of subcellular organelles and transporters was higher in the sprouting bud library while those related with antioxidant, translation regulator and response to stimulus showed a greater percentage in the dormant bud library [8]. Dormancy-related genes were identified by the analysis of dormant bud (Banjhi) specific transcriptome of tea (*Camellia sinensis* (L.) O. Kuntze) from a cDNA library [7]. The expression pattern of tea miRNAs in active and dormant bud were analyzed, and the role of target transcripts regulated by these miRNAs in relation to bud dormancy was discussed, but the molecular mechanism of tea plant dormancy remains unclear [4]. Apart from the studies related to dormancy, some reports have been focused on the adaptation of tea plants to cold environment, low temperature, and low light stress [3, 9]. Analysis of global transcriptome profiles of *Camellia sinensis* during cold acclimation showed that the “carbohydrate metabolism” and “calcium signaling” pathways might play a vital role in tea plants’ responses to cold stress [3]. However, the molecular mechanism governing the growth of buds and leaves of the evergrowing cultivar in winter is not known. So far, few studies have specifically reported on the growth of tea buds in winter, and only one closely-related report was about peach dormancy found in Mexico. Peach dormancy study reported the continuous growth of an evergrowing peach mutant in short-period light and low temperature [10–12]. The gene responsible for the evergrowing genotype was a dormancy-associated MADS-box (DAM) transcription factor. The DAM gene is expressed in wild-type peach trees, whereas it becomes silent in the mutant. The DAM gene is a member of the MADS-box family. Overexpression of another MADS-box gene, BpMADS4, prevents dormancy in *Populus tremula* [13].

The physiological and molecular differences between the winter tender shoots and spring tender shoots are the key to understanding the mechanism of development of winter tea tender shoots. Differences in gene expression leading to variation in the levels of their corresponding proteins directly affect the physiological processes in plants [14]. The differentially accumulated proteins detected by proteomic techniques between different tissues and organs under various stages and conditions reveal the molecular mechanisms involved in the developmental regulation, signal transduction, and metabolism. Proteomics is an invaluable tool for analyzing the differentially accumulated proteins under different environmental stimuli; however, this technology has not been fully exploited to elaborate the physiological and molecular differences between the various tea traits [15–18].

We conducted the present study to identify the differentially accumulated proteins between winter and spring tender shoots of the evergrowing tea cultivar “Dongcha11” through proteomics methods. Our study will provide novel insights into the physiological mechanism of tea tender shoots in response to low temperature and dim light stress.

## Methods

### Plant material

A tea tree plant which could grow tender shoots in winter was selected from a landrace tea population by Professors Jiaxian Li and Hualin Huang of Tea Research Institute, Guangdong Academy of Agricultural Sciences. The special individual plant was multiplied vegetatively by single node cuttings and became a clonal tea cultivar “Dongcha11”. Dongcha11 were planted in Yingde (24.30° N and 113.39° E, lateritic red soil,pH 5.86,suitable for tea tree growth). Regular tea went to winter dormancy at the end of January 2013, whereas Dongcha11 continued to generate new tender shoots. Winter tender shoots were collected from Dongcha11 on 23rd January 2013, and spring tender shoots were collected from Dongcha11 on 2nd April 2013 respectively. Harvested samples were flash frozen in liquid nitrogen and stored in a − 80 °C freezer until further use.

### Methods

#### Tea protein extraction and content determination

Protein extractions were performed as previously described [16] with minor modifications, sampling was done with three biological replicates. Briefly, 2 g tea winter tender shoots or spring tender shoots were randomly collected from Dongcha11 respectively and individually prepared for protein extraction, and each tea tender shoot sample was ground to a fine powder with 0.2 g PVPP in liquid nitrogen in a pre-cooled mortar. The powders were mixed with ice-cold TCA/acetone (containing 1% (*v*/v) β-mercaptoethanol) and transferred into a 10 mL eppendorf tube. After centrifugation at 10,000×*g* for 10 min at 4 °C, the pellet was washed twice with ice-cold acetone and dried in vacuum at 4 °C for 5 min. Then, phenol (containing 0.5% (*w*/*v*) DTT) was added to the pellet and the mixture was shaken vigorously at 4 °C for 30 min to facilitate the dissolution of proteins in phenol. The phenol solution was clarified by centrifugation at 12,000×*g* for 10 min at 4 °C. The pellet was re-extracted using the same procedure and the supernatants were combined together. Proteins were precipitated by adding five volumes chilled methanol containing 0.1 M ammonium acetate to the pooled supernatants and incubating overnight at −20 °C. The precipitated proteins were collected by centrifugation at 12,000×*g* for 10 min at 4 °C. The protein pellet was washed twice with pre-cooled methanol and once with acetone, and then vacuum-dried at 4 °C for 5 min. The resulting protein powder was stored at −80 °C for future use. The protein concentration was determined by Bradford assay.

#### Tea protein two-dimensional electrophoresis (2-DE) and image analysis

For 2-DE, a total of 1.5 mg protein was first subjected to isoelectric focusing (IEF) and then separated by 2nd dimension SDS-PAGE. First-dimension IEF was conducted using pH 3–10 NL IPG strips (ReadyStrip, 24 cm, Bio-Rad, USA). The strips were rehydrated in a rehydration solution containing the protein sample for 12 h at 20 °C. IEF was performed on Protean® IEF Cell (Bio-Rad) under voltage conditions ramping to 10,000 V within 8 h, and then at 10,000 V until a total of 100–160 kVh was reached.

After IEF, the strips were equilibrated immediately for 15 min in equilibration buffer 1 (50 mM Tris-HCl, pH 8.8, 6 M urea, 30% (*v*/v) glycerol, 4% (*w*/*v*) SDS, 2% (w/v) DTT, 0.001% (w/v) bromophenol blue), then 15 mL equilibration buffer 2 (50 mM Tris-HCl, pH 8.8, 6 M urea, 30% (v/v) glycerol, 4% (w/v) SDS, 2.5% (w/v) iodoacetamide, 0.001% (w/v)) was added and incubated for 15 min. Second dimension SDS-PAGE was performed in a 12.5% (v/v) polyacrylamide-SDS gel (Protean® Plus Dodeca Cell, Bio-Rad). After electrophoresis, the gels were stained with Coomassie Brilliant Blue. Stained 2-DE gels were scanned with GS-800 Calibrated Densitometer (Bio-Rad), and data were analyzed by PDQuest software version 8.0 (Bio-Rad) as described by the manufacturer. The spots were detected by the software automatically and then subjected to careful manual editing and confirmation. Each spot included on the standard gel met the following criteria: it was present in at least two of the three gels and was qualitatively consistent in size and shape in the replicate gels. The relative volume of each spot was assumed to represent its expression level. After manual examination, the volume of each well-separated spot was compared between winter and spring tender shoots to identify differentially accumulated protein spots. The statistical significance of the quantitative data was determined using a Student’s *t*-test. A spot abundance ratio of greater than 2.0 (*p* <0.05) (a spot present uniquely or present in two-fold abundance in one sample relative to the other) was used as the threshold for a protein being differentially accumulated in subsequent studies.

#### Mass spectrometry (MS) analysis for protein identification

Differentially expressed protein spots were excised and digested with trypsin (Promega, Madison, WI, USA). Gel slices were placed in 0.1 M NH_4_HCO_3_ in 30% (*v*/v) acetonitrile (ACN), vortexed, and then destained for 15 min. The gel pieces were washed twice with ACN and immersed in 5 μl 2.5–10 ng/μL trypsin for 30–60 min at 4 °C. Trypsin-saturated gel pieces were depleted of excessive enzyme solution and digested in 20 μL 25 mM NH_4_HCO_3_ (pH 7.8–8.0) at 37 °C for 20 h. The peptides were extracted two times with 100 μL 0.1% (v/v) TFA in 60% (v/v) ACN. Extracts were pooled together and lyophilized. Lyophilized peptides were reconstituted with 3 μL 0.1% (v/v) trifluoroacetic acid (TFA) in 30% (v/v) ACN. The resulting peptides were subjected to MALDI-TOF/TOF MS analysis (Ultraflextreme, Bruker, Bremen, Germany). Peptide mass data was analyzed using MASCOT (Matrix Science, London, UK). The acquired data were analyzed by searching the SwissProt and/or NCBI green plant database for identified proteins with Mascot software. The search parameters allowed for one missed cleavage, fixed modifications of pyridylethyl, and variable modifications of methionine oxidation and cysteine carboxyamidomethylation with a peptide mass tolerance of 100 ppm. The main criteria used for accepting an identified protein should be *p* <0.05.

#### Quantitative RT-PCR analyses

Total RNA was extracted from winter tender shoots or spring tender shoots individually using 1 mL of TRIzol reagent (Invitrogen). For all samples, 1 μg of total RNA was converted to cDNA using PrimeScript® 1st Strand cDNA Synthesis Kit (Takara) according to the manufacturer’s instructions. Quantitative real-time PCR was performed with the SYBR® Premix Ex Taq kit (Takara) and a Stratagene Mx3000P real-time PCR instrument (Agilent) using three-step cycling conditions of 95 °C for 5 min followed by 40 cycles of 95 °C for 10 s, 58 °C for 20 s and 72 °C for 20 s. The reaction mixture (20 μL) contained 1 μL of cDNA solution, 10 μL SYBR® Premix Ex Taq and primers at a concentration of 6 μM each. All gene-specific primers (Additional file 1: Table S1) were designed using the Primer Version 5.0 (PREMIER Biosoft International) and were based on the cDNA sequences. The tea gene ubiquitin (UBI) was used as a reference for calculating relative transcript abundance. The primers are: forward (5′-CAGGACAAAGAGGGCATACC-3′) and reverse (5′-CACGCAATCGGAGAACCAAG-3′). The relative quantification of RNA expression was calibrated using formula 2^-ΔΔCt^ method. Samples of winter tender shoots and spring tender shoots were three biological replicates with three technical replicates.

#### Biochemical measurements

Tea sample production and processing methods refer to “Descriptors and Data Standard for Tea (*Camellia spp.*)” approach [19]. Moisture measurement was referred to GB / T 8304-2002 using the 103 °C constant weight method. Water extracts were measured according to GB / T 8305-2002. Determination of polyphenols was referred to GB / T 8313-2008. Determining the contents of free amino acids was referred to GB / T 8314-2002, and soluble sugars were carried out as previously described [20].

## Results

### Comparison of proteomic profiles and identification of differentially accumulated proteins between winter and spring tender shoots

**Fig. 1:**
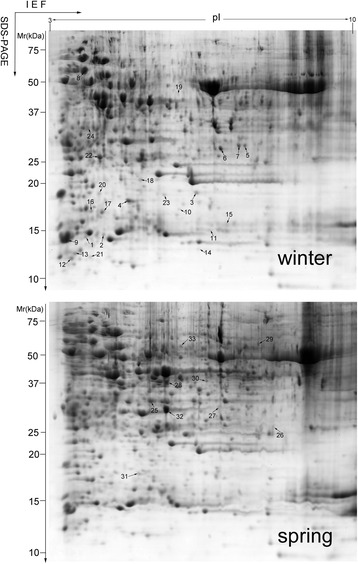
2-DE image analysis of tea proteins between winter and spring tender shoots of the evergrowing tea tree Dongcha11. This 2-D gel is a representative image of the Commassie Brillant Blue stained gel with three biological replicates. Differentially accumulated protein spots are marked with corresponding numbers, including 24 proteins with increased abundance (upper panel) and nine with decreased abundance (lower panel) in winter tender shoots as compared with the spring shoots. Identified proteins are listed in Tables 1 and 2

We identified 42 protein spots on the 2-DE gel images that accumulated differentially (more than 2-fold in abundance) between the winter and spring tender shoots (*p* <0.05) with the MALDI-TOF/TOF MS analysis. From these protein spots, 36 differentially accumulated proteins were retrieved from the SwissProt and NCBInr databases by Mascot analysis. Among them, three out of 36 differentially accumulated proteins were identified as a mixture of multiple proteins. Therefore, these proteins were excluded from the further analysis. The remaining 33 proteins included 24 proteins with increased abundance (Table 1) and nine with decreased abundance (Table 2) in winter tender shoots as compared with the spring shoots (Fig. 1, Additional file 2: Table S2).

**Table 1:** Proteins with increased abundance in “Dongcha11” shoots newly germinated in winter compared to spring tender shoots

Spot No.	Protein name	Species	Accession	Ratio	Mascot score	MS coverage%	Theoretical Mr.(kDa)/pI	Expressed Mr.(kDa)/pI
Photosynthesis								
1	Ribulose 1,5-bisphosphate carboxylase	*Aralidium pinnatifidu*	AAG24624	>100	80	5	52.13/6.2	14.46/4.07
2	Ribulose 1,5-bisphosphate carboxylase	*Aralidium pinnatifidu*	AAG24624	75.93	76	5	52.13/6.2	14.74/4.46
3	Ribulose bisphosphate carboxylase large chain	*Acacia farnesiana*	P93998	19.9	415	12	50.86/6.2	20.22/6.00
4	Ribulose bisphosphate carboxylase large chain	*Acacia farnesiana*	P93998	13.38	427	7	50.86/6.2	18.93/5.03
5	Ribulose bisphosphate carboxylase large chain	*Akania bidwillii*	Q07281	5.62	126	8	52.31/6.2	29.14/6.82
6	Ribulose bisphosphate carboxylase large chain	*Akania bidwillii*	Q07281	3.81	355	13	52.31/6.2	29.21/6.73
7	Ribulose bisphosphate carboxylase large chain	*Begonia metallica X sanguinea*	P28383	3.02	308	15	49.17/6.6	28.32/6.43
8	RuBisCO large subunit-binding protein subunit alpha, chlorop	*Brassica napus (Rape)*	P21239	2.09	344	8	57.71/4.7	59.74/4.07
9	Plastocyanin	*Capsella bursa-pastoris*	P00294	2.51	65	24	10.43/4.1	14.11/3.54
10	ATP synthase delta chain, chloroplastic	*Spinacia oleracea*	P11402	5.77	47	6	27.66/5.7	17.54/5.76
Cell structure								
11	Putative histone H3-like 5	*Vitis vinifer*	Q9FKQ3	>100	52	14	15.64/11.9	15.19/6.22
12	Profilin-1	*Ricinus communis*	O82572	2.09	84	29	14.31/4.4	12.31/3.61
13	Profilin-A	*Camellia sinensis*	Q9FUD1	2.75	71	9	14.35/4.8	12.72/3.73
14	Histone H4	*Arabidopsis thaliana*	P59259	>100	42	13	11.40/12.0	13.24/6.04
15	Histone H2A.1	*Triticum aestivum*	P02275	>100	52	8	15.58/11.1	16.08/6.49
Protein synthesis & destination								
16	Predicted protein	*Populus trichocarpa*	XP_002301464	6.5	145	9	29.47/10.0	17.32/4.19
17	Predicted protein	*Populus trichocarpa*	XP_002301464	2.09	161	10	29.47/10.0	17.21/4.42
18	Proteasome subunit beta type-6	*Arabidopsis thaliana*	Q8LD27	3.89	51	6	25.19/5.2	22.22/5.23
19	Eukaryotic initiation factor 4A-1	*Oryza sativa Japonica*	P35683	>100	59	9	47.34/5.3	45.58/5.79
Transporters								
20	Hypothetical protein	*Camellia sinensis*	AEC10968	6.97	99	8	24.67/10.4	19.54/4.36
21	Phosphatidylglycerol/phosphatidylinositol transfer protein p	*Camellia sinensis*	AEC10983	11.57	139	25	16.96/4.8	12.47/4.15
Disease/defense								
22	Putative In2.1 protein	*Triticum aestivum*	CAA76758	9.94	56	4	27.25/5.3	26.48/4.35
23	Peptide methionine sulfoxide reductase	*Solanum lycopersicum*	P54153	10.56	88	7	22.25/6.1	19.72/5.61
Protein of unknown functions								
24	unknown	*Picea sitchensis*	ADE77382	3.53	91	4	37.84/5.8	32.90/4.11

Only protein spots that changed in abundance at least 2-fold in at least two of three replicates are included

Ratio: Protein abundance in winter shoots/ Protein abundance in spring shoots

**Table 2:** Proteins with decreased abundance in “Dongcha11” shoots newly germinated in winter compared to spring tender shoots

Spot No.	Protein name	Species	Accession	Ratio	Mascot score	MS coverage%	Theoretical Mr.(kDa)/pI	Expressed Mr.(kDa)/pI
Metabolism /Sugars and polysaccharides								
25	Fructokinase	*Actinidia eriantha*	P37829	0.45	45	3	33.97/5.4	32.18/5.16
26	Phosphomannomutase	*Nicotiana tabacum*	Q1W375	0.05	37	7	28.79/5.7	26.47/7.17
27	Eukaryotic galactinol synthase	*Camellia sinensis*	AFR79417	0.19	59	6	33.78/6.0	30.87/6.27
Secondary metabolism								
28	Flavonol synthase	*Camellia sinensis*	ABM88786	0.46	518	29	37.60/5.5	38.57/5.42
29	Bifunctional 3-dehydroquinate dehydratase/shikimate dehydrog	*Arabidopsis thaliana*	Q9SQT8	0.12	58	1	66.10/6.4	58.43/6.93
Disease/defense								
30	Monodehydroascorbate reductase	*Camellia sinensis*	ACH87167	0.46	53	3	47.37/6.0	38.99/6.08
31	PREDICTED: protein lin-28 homolog	*Brachypodium distachyon*	XP_003562975	<0.01	58	13	19.64/5.9	18.47/4.96
Protein of unknown functions								
32	Unknown	*Picea sitchensis*	ABK23421	0.49	70	5	42.93/9.5	30.04/5.41
33	Predicted protein	*Populus trichocarpa*	XP_002298565	0.41	86	4	56.61/5.8	57.00/5.66

Only protein spots that changed in abundance at least 2-fold in at least two of three replicates are included

Ratio: Protein abundance in winter shoots/ Protein abundance in spring shoots

The remaining 33 identified protein spots corresponded to 29 unique differentially abundant proteins because several spots represented identical proteins (spots 1 and 2, spots 3 and 4, spots 5 and 6, spots 16 and 17). Among the 29 unique proteins, 26 had known functions, whereas three had a predicted or unknown function.

### Functional classification of identified proteins was distinctly different between winter and spring tender shoots

We categorized the 33 differentially accumulated proteins between winter and spring tender shoots into eight groups based on their potential physiological functions or involvement in different biological processes as described [15, 16] such as photosynthesis, cell structure (cytoskeleton and chromosomes), protein synthesis & destination, transporters, sugars and polysaccharides, secondary metabolism, disease/defense, and protein of unknown functions (Tables 1 and 2).

The majority of differentially accumulated proteins (spots 1-10) were associated with photosynthesis followed by a cellular structure with five spots (including two cytoskeletal proteins (spots 12 and 13), and three chromosome proteins (spots 11, 14 and 15). Four protein spots (spots 22, 23, 30 and 31) were identified as disease resistant/defense proteins. Four other protein spots (spots 16, 17, 18 and 19) were in the protein synthesis category, and three (spots 25, 26 and 27) in metabolism/sugars and polysaccharides. Secondary metabolism proteins were mapped to 2 proteins spots (spots 28 and 29) (Fig. 1; Tables 1 and 2).

**Fig. 2:**
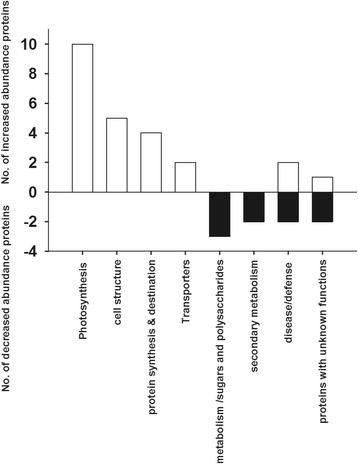
The histogram represents the numbers of differentially increased and decreased abundance proteins in winter tender shoots in each functional category compared to spring tender shoots □ No. of increased abundance proteins: Numbers of differentially increased abundance proteins in winter tender shoots, 24 protein spots were categorized into six functional groups. ■ No. of decreased abundance proteins: Numbers of differentially decreased abundance proteins in winter tender shoots, nine protein spots were categorized into four functional groups

The differentially increased abundance proteins in winter tender shoots showed distinct differences in biological functions (Fig. 2). All identified proteins involved in photosynthesis, cell structure, and protein synthesis and destination were increased abundance in winter tender shoots. Contrastingly, differentially decreased abundance proteins in winter tender shoots were mainly involved in metabolism (sugars and polysaccharides) and secondary metabolism processes.

Some disease/defense-proteins had increased abundance in winter tender shoots, and others had increased abundance in spring tender shoots. The protein expression of peptide methionine sulfoxide reductase (spot 23) and putative In2.1 protein (spot 22) significantly increased in winter tender shoots, while protein lin-28 homolog (spot 31) accumulated more than 100-fold higher in spring tender shoots over in winter tender shoots.

### Changes in transcript abundance of differentially accumulated proteins between winter shoots and spring shoots

**Fig. 3:**
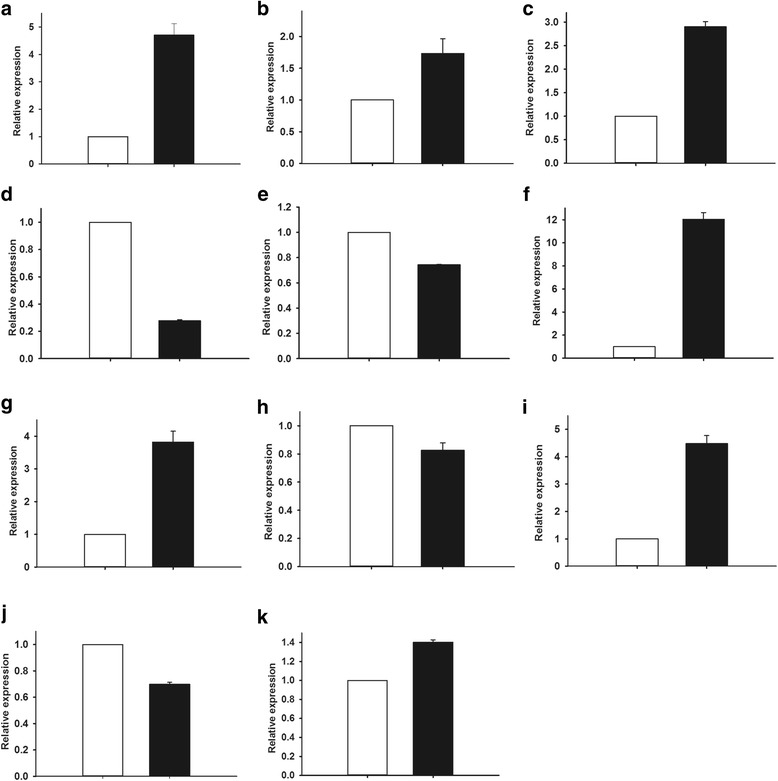
Relative RNA levels of selected genes for corresponding differentially accumulated proteins by real-time RT-PCR between spring tender shoots and winter tender shoots. **a**: Ribulose 1,5-bisphosphate carboxylase; **b**: ATP synthase delta chain; **c**: Profilin-A; **d**: Histone H4; **e**: Eukaryotic initiation factor 4A-1; **f**: Proteasome subunit beta type-6; **g**: Putative In2.1 protein; **h**: Fructokinase partial; **i**: Eukaryotic galactinol synthase; **j**: Bifunctional 3-dehydroquinate dehydratase; **k**: Monodehydroascorbate reductase. Data are presented as the mean ± SE of three biological replicates. □ Spring tender shoots ■ Winter tender shoots

To validate the expression of the identified proteins at the transcriptional level, we analyzed the differences in mRNA abundance between winter and spring tender shoots. We selected 11 genes from different functional categories for quantitative real-time PCR (qRT-PCR) analysis. These included: Ribulose 1,5-bisphosphate carboxylase, ATP synthase delta chain, Proteasome subunit beta type-6, Profilin-A, Eukaryotic initiation factor 4A-1, Histone H4, Putative In2.1 protein, Bifunctional 3-dehydroquinate dehydratase, Eukaryoticgalactinol synthase, Monodehydroascorbate reductase, and Fructokinase (Fig. 3).

Among them, six differentially accumulated proteins showed similar changes in their mRNA expression such as proteins involved in photosynthesis (ribulose 1,5-bisphosphate carboxylase, ATP synthase delta chain), protein synthesis and destination (proteasome subunit beta type-6), cytoskeleton (Profilin-A), disease/defense (putative In2.1 protein), and secondary metabolism (Bifunctional 3-dehydroquinate dehydratase) (Fig. 3 and Tables 1 and 2).

However, alteration in protein expression levels does not always correlate well with the changes in mRNA levels. In this study also, we observed discrepancies in the abundance of protein and mRNA, for example, Eukaryoticgalactinol synthase, monodehydroascorbate reductase, fructokinase, Eukaryotic initiation factor 4A-1, Histone H4*.* Moreover, the abundance of histone H4 increased in winter tender shoots while their mRNA expression decreased in winter tender shoots, while fructokinase accumulation was high in spring tender shoots but its mRNA expression showed no significant changes between shoots harvested in winter and spring. The inconsistency between mRNA levels and protein levels can be attributed to post-transcriptional, translational, and post-translational regulation of gene expression. However, their modification to play a functional role in the shoots of the evergrowing Dongcha11 required further study.

### Analysis of biochemical components between winter shoots and spring shoots

**Fig. 4:**
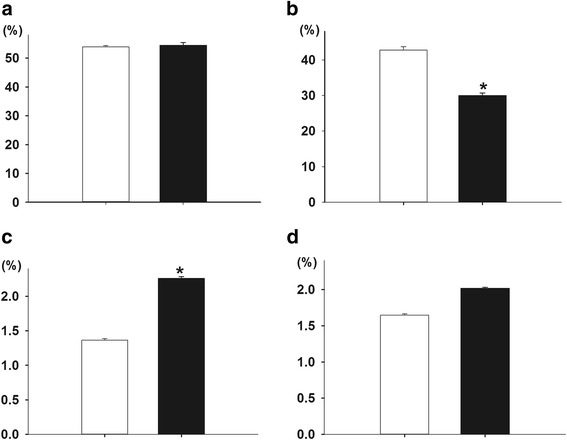
The contents of water extracts, tea polyphenol, soluble sugar contents in winter shoots and spring shoots of the evergrowing tea tree Dongcha11. Different letters indicate groups with significant differences at *p* = 0.05 (least significant difference--*), data are presented as the mean ± SE of three replicates. **a**: Water extracts; **b**: Tea polyphenols; **c**: Soluble sugars; **d**: Free amino acids. □ Spring tender shoots ■ Winter tender shoots

Water extracts (aqueous extract) between spring tender shoots and winter tender shoots did not show significant differences (Fig. 4). The freshness and the processed quality of the winter shoots are similar to that of spring shoots. The winter tender shoots contained a lower polyphenol content (29.98%) as compared with spring tender shoots (42.75%) (Fig. 4). This data was consistent with the finding that differentially accumulated proteins involved in secondary metabolism were decreased abundance in winter tender shoots in proteomic analysis. Total soluble sugar and amino acids contents were higher in winter tender shoots as compared with spring tender shoots (Fig. 4). The specific types of soluble sugars and/or amino acids in winter tender shoots require further research to determine the exact biological processes involved in the evergrowing phenotype in winter.

## Discussion

### Differentially accumulated proteins involved in photosynthesis increased significantly in winter tender shoots

All differentially accumulated proteins involved in photosynthesis showed dramatically increased abundance in winter tender shoots. These proteins are mainly associated with the dark reaction in photosynthesis (ribulose-1,5-bisphosphate carboxylase, ribulose-l,5-bishosphatecarboxylase/oxygenase large subunit (RBCL), ribulose-l,5-bishosphate carboxylase/oxygenase large subunit-binding protein subunit alpha), photosynthetic phosphorylation pathway (plastocyanin), and chloroplast ATP synthase β chain. Our qRT-PCR results demonstrated that these proteins showed a positive correlation between the transcriptional level and the protein level.

Previous studies have shown that low temperature and weak light caused blockage of plant electron transfer, decrease in carbon assimilation enzyme activity, and photosynthetic ability, and this adverse condition is an important cause for restriction in crop production [21]. Ribulose-l,5-bishosphate carboxylase/oxygenase is a key enzyme catalyzing the photosynthetic carbon cycle and the first reaction in photorespiration. Therefore, this enzyme plays an important role in regulating the rate of photosynthesis. Low temperature and dim light stress decrease Rubisco activase (RCA), Rubisco activity, and gene expression causing a decrease in net photosynthetic rate (Pn) in cucumber seedlings [22].

In our study, the protein abundances of Rubisco and electron mediator plastocyanin (PC) in photosynthetic phosphorylation pathway and ATP synthase delta chain dramatically increased in winter tender shoots. This abundance could be attributed to the adaption of Dongcha11 to low temperature and light stress. Plant photosynthetic efficiency declines in winter in response to low temperature and light. Dongcha11 probably increases the expression of photosynthesis proteins to maintain the normal physiological activity of evergrowing leaves in winter.

### Proteins involved in cell structure increased significantly in winter tender shoots

Our data showed that several cytoskeleton-associated proteins were significantly increased abundance in winter tender shoots, which was consistent with their mRNA expression (e.g., profilin-1 and profilin-A). Interestingly, phosphatidylinositol transfer protein (PITP) was also significantly increased abundance in the winter tender shoots.

Actin is a key player in the microfilament skeleton and maintains a dynamic balance between polymerization and depolymerization; thus, they can rapidly react to the stimulation from endogenous cellular factors or exogenous signals to ensure cellular homeostasis. A series of actin-binding proteins are involved in actin polymerization and depolymerization process [23]. As the first identified monomeric actin-binding protein, profilin is distributed widely in fast-growing root hairs and pollen tubes, and its activity is closely associated with pollen tube growth and root hair occurrence [24]. Arabidopsis seedlings deficient in profilin show abnormal development [23].

Profilin has binding sites with its ligand phosphatidylinosital biphosphate (PIP2) [25] and is located in the protruding parts of the root hair cells [26]. PITPs are capable of binding with phosphatidyl inositol (PI) or phosphatidylcholine and facilitate their transfer between intracellular membrane components [27, 28]. PITPs regulate the intercellular membrane transport system and phospholipid signal transduction playing a primary role in response to stress and regulation of development in higher plants [29]. In fact, PITPs function in tea tree dormancy [7]. Therefore, our result indicated that profilin and PITP may act synergistically through PIP2 to overcome the stress of low temperature and weak light in the evergrowing tea tree.

Histones are also associated with cell structure, and the abundance of Histone-like 5, Histone H4, and Histone H2A.1 was significantly increased in winter tender shoots. Post-translational modification of histone is closely related to chromosome remodeling and chromosome function, and also plays an important role in cell fate determination, cell growth, and other biological processes [30, 31]. Further, histone is essential to both dormancy and release of dormancy. Santamaría et al. found that chestnut genomic DNA methylation and histone H4 acetylation are closely associated with bud dormancy formation and release [32]. Fang et al. cloned cold resistant related H1-histone gene in tea [33]. It is noteworthy that in our study, differentially accumulated histone proteins showed a higher abundance in winter tender shoots while their mRNA transcripts were higher in their spring counterparts. This discrepancy may be due to a complicated regulatory role of histone involved in evergrowing in winter.

### Proteins involved in protein synthesis increased significantly in winter tender shoots

Four differentially accumulated proteins including eukaryotic translation initiation factor 4A (eIF4A), proteasome subunit beta type-6, and 2 predicted proteins were increased abundance in winter tender shoots.

Previous studies identified that eIF4A is an RNA-binding protein and functions as RNA helicase. EIF4A, eIF4E, and eIF4G form an eIF4F complex. This complex regulates the initiation of eukaryotic translation [34, 35]. Further studies showed that besides initiation of protein synthesis, eIF4A plays a key role in a series of biological processes such as signal transduction. eIF4A negatively regulates Dpp/BMP signaling in a translation-independent manner to promote Mad and Medea degradation in the SMAD complex during Drosophila embryo development [36]. Recent studies revealed that as a translation initiation factor, eIF4A, interacts with an oncogenic protein, thus, inhibiting the formation of eIF4F complex and negatively affecting the interaction of eIF4E-eIF4G and/or blocking the function of BRAF (V600) [37, 38]. The expression level of eIF4E in the eIF4F complex is correlated to that of matrix metalloproteinase MMP-9 [39, 40]. In contrast, the function of eIF4A is not well-studied in plants. It was previously considered to function in response to abiotic stress and help improve crop yields under stress conditions [41]. Our finding that the protein abundance of eIF4A and protease subunits dramatically increased in winter tender shoots suggested their role in helping the evergrowing tea tree to overcome the environmental stress and maintaining growth and development in winter.

### Proteins involved in metabolism and secondary metabolism decreased significantly in winter tender shoots

Compared to spring tender shoots, the differentially accumulated proteins related to carbohydrate metabolism (e.g. fructokinase, phosphomannomutase and eukaryotic galactinol synthase) significantly decreased in winter tender shoots.

Fructokinase is the first enzyme in the glycolytic pathway. As a key enzyme of fructose catabolism, fructokinase can also serve as a fructose sensor and a signaling molecule in plants. Therefore, it can influence metabolism and growth by regulating the plant life cycle [42].

Phosphomannomutase is a key enzyme in mannose metabolism that catalyzes the conversion of mannose-1-phosphate to mannose-6-phosphate. Both fructokinase and phosphomannomutase are involved in the synthesis of vitamin C (ascorbic acid) in plants. Monodehydroascorbate reductase (MDHAR) plays an important role in the regeneration of vitamin C (ascorbic acid). Ascorbic acid is oxidized to monodehydroascorbate (MDHA) by ascorbate peroxidase (APX). MDHA is highly unstable and is re-converted into ascorbic acid by MDHAR action [43].

Galactinol synthase functions in the first step of raffinose oligosaccharide family (RFO) synthesis, which is a key step for the synthesis and accumulation of RFO family members. RFO provides a carbon source for photosynthesis in plants growing at low temperature and also serves as a supplemental carbon source when starch metabolism in plants is suppressed under low temperatures [44]. However, the mechanism of galactinol synthase involved in the growth of winder tender shoots required further investigation.

In plants, synthesis of various phenolic compounds may depend on the shikimate pathway, phenylpropanoid pathway, and flavonoid biosynthetic pathway. Shikimate pathway is the main bridge between glucose metabolism and polyphenol metabolism. In the winter tender shoots, protein abundance of shikimate dehydrogenase (SDH) and dehydrogenation quinic acid (DHQ), and flavonol synthase of flavonoid biosynthesis pathway significantly decreased. In most microorganisms, DHQ and SDH are enzymes with a single function, but in plants, they may be fused to form a bifunctional enzyme. The advantage of DHQ-SDH compound in the shikimate pathway is that it can restrict the formation of intermediates; and thus, increases the turnover rate of metabolites [45]. In the flavonoid biosynthetic pathway, flavonol synthase (FLS) catalyzes flavanonols into flavonols [14, 46]. Both shikimate and flavonoid pathways regulate plant development and protect plants from UV and stress damage, which may contribute to the unique quality of specific plants. The shade treatment shows specific effects on different flavonoids of fresh tea leaves [47]. The specific effects include a reduction in the polymerization of catechins and glycosylation of flavonols and significant decrease expression of genes related to flavonoids synthesis such as PAL, F3H, F3’H, DFR, ANR, and UFGT. Moreover, flavonoid biosynthesis often increases in response to external stress factors such as light and cold temperature [48, 49]. In our study, DHQ-SDH and FLS were of low abundance in the winter tender shoots. Biochemical analysis showed that the polyphenol content of spring tender shoots was significantly higher than their winter counterparts, which is in agreement with previous studies that light intensity impacts the synthesis of polyphenol secondary metabolites in plants [14].

## Conclusion

Our study shows that differentially accumulated proteins between winter and spring shoots displayed specific functional classifications. Rubisco, and electron mediator PC and ATP synthase protein contents significantly increased in winter tender shoots indicating their role in maintaining normal growth under low temperatures and light conditions. An increase in other proteins such as eIF4A, protease subunits, phosphatidylinositol transfer protein (PITP), and profilin in winter tender shoots suggests that they might help in the development process of winter buds. The decrease in abundance of proteins involved in secondary metabolism corroborated the low content of tea polyphenols in winter.

To our knowledge, this is the first application of proteomics to reveal physiological and molecular characteristics of winter growing shoots of tea trees. Further research will combine non-gel proteomics with transcriptomic analysis to further analyze the expression patterns of the differentially accumulated proteins.

## Additional files

Additional file 1: Table S1. Primers used in real-time RT-PCR for genes of differentially accumulated proteins in winter shoots and spring shoots. (DOC 23 kb)

Additional file 2: Table S2. Mascot identification data of the differentially accumulated proteins between winter and spring tender shoots of the evergrowing tea tree Dongcha11. (XLS 521 kb)

## Abbreviation

2-DE: Two-dimensional electrophoresis
ACN: Acetonitrile
ADF: Actin-depolymerizing factor
APX: Ascorbate peroxidase
DAM: Dormancy-associated MADS-box
DHQ: Dehydrogenation quinic acid
eIF4A: Eukaryotic translation initiation factor 4A
FLS: Flavonol synthase
GS: Galactinol synthase
IEF: Isoelectric focusing
MALDI-TOF / TOF MS: Matrix-assisted laser desorption ionization time of flight mass spectrometry
MDHA: Monodehydroascorbate
MDHAR: Monodehydroascorbate reductase
PC: Plastocyanin
PI: Phosphatidyl inositol
PIP2: Phosphatidylinositol bisphosphate
PITP: Phosphatidylinositol transfer protein
PMM: Phosphomannomutase
RBCL: Ribulose-l,5-bishosphatecarboxylase/oxygenase large subunit
RCA: Rubisco activase
RFO: Raffinose oligosaccharide
SDH: Shikimate dehydrogenase
