# Supplementary material for: Comparative proteomics reveals the physiological differences between winter tender shoots and spring tender shoots of a novel tea (Camellia sinensis L.) cultivar evergrowing in winter
Source: BMC Plant Biol. 2017 Nov 20;17:206. doi: 10.1186/s12870-017-1144-x (PMC5697017; doi:10.1186/s12870-017-1144-x)
Supplement: Supplementary file 1 — Primers used in real-time RT-PCR for genes of differentially accumulated proteins in winter shoots and spring shoots. (DOC 23 kb) [file 12870_2017_1144_MOESM1_ESM.doc]

Additional file 1: Table S1

| Spot no. ^a^ | Protein name | Accession no. | Primer sequence(5' to 3') | |
| --- | --- | --- | --- | --- |
|  |  |  | Forward | Reverse |
| 1,2 | Ribulose 1,5-bisphosphate carboxylase | AAG24624 | GTGAACTCCCAACCATTT | TTTCAGCCTGTGCTTTAT |
| 10 | ATP synthase delta chain | P11402 | TCTTCGAAGCTGCCAAGGTT | CGTAAATTCCACGGGTGGCT |
| 18 | Proteasome subunit beta type-6 | Q8LD27 | AAGACCATCCTGCAAAGCGA | CTCCGCTCTGTCGCCATATT |
| 13 | Profilin-A | Q9FUD1 | CTGAGCCGGGTTATTTGGCT | CAGTAACACCACCAGGTCCC |
| 19 | Eukaryotic initiation factor 4A-1 | P35683 | GGCTATAGCAGTAGCCGTGG | GCTGCACACCCACATAGAGT |
| 14 | Histone H4 | P59259 | GGCAGGGGAGGACTCTCTAT | ACAACCCCCAAACCTACCTT |
| 22 | Putative In2.1 protein | CAA76758 | AAGGAGAAAGTTTACCCGCAGA | TCCAGACTCTCTCCCTTGAC |
| 29 | Bifunctional 3-dehydroquinate dehydratase | Q9SQT8 | CACGCCAAAAGATACCAGGC | CAAAGCCTTGGCGGATCAAC |
| 27 | Eukaryotic galactinol synthase | AFR79417 | ATATGGCCCTAAACTCGCCC | CCACCACAAGAGGGTAAGCA |
| 30 | Monodehydroascorbate reductase | ACH87167 | TGGCAAAGGATGGTCTCACC | ATACCAACATCGCCAAGGCT |
| 25 | Fructokinase | P37829 | TTCCGTGGATGTGTGGATGG | GCACCAACAAACGAGTCACC |
| Internal standard | ubiquitin (UBI) |  | CAGGACAAAGAGGGCATACC | CACGCAATCGGAGAACCAAG |

^a^ Consistent with the spot numbers in Table 1 ,2 and Fig. 1.
